# Community living causes changes in metabolic behavior and is permitted by specific growth conditions in two bacterial co-culture systems

**DOI:** 10.1128/jb.00075-25

**Published:** 2025-05-14

**Authors:** Elizabeth Ellis, Sam Fulte, Skyler Boylan, Alaina Flory, Katherine Paine, Sophia Lopez, Grace Allen, Kanwar Warya, Javier Ortiz-Merino, Sadie Blacketer, Samantha Thompson, Sierra Sanchez, Kayla Burdette, Audrey Duchscherer, Nick Pinkham, Joseph D. Shih, Lilah Rahn-Lee

**Affiliations:** 1 Department of Biology, William Jewell College 4536, Liberty, Missouri, USA; 2 Department of Microbiology and Cell Biology, Montana State University 123776 , Bozeman, Montana, USA; University of Massachusetts Chan Medical School, Worcester, Massachusetts, USA

**Keywords:** co-culture, microbial communities, transcriptomics

## Abstract

Although bacteria exist in complex microbial communities in the environment, their features and behavior are most often studied in monoculture. While environmental enrichments or complex co-cultures with tens or hundreds of members might more accurately represent the natural communities of bacteria, we sought to create simple pairs of organisms to learn what conditions create successful co-culture and how bacteria change transcriptionally when a partner species is present. We grew two pairs of organisms in co-culture, *Pseudomonas aeruginosa* and *Escherichia coli* and *Lacticaseibacillus rhamnosus* and *Bacteroides thetaiotaomicron*. At first, both co-cultures failed, with one organism outcompeting the other. However, through manipulating media and environmental conditions, we created co-cultures with stable member ratios over many generations for each community. We then show that changes in the expression of metabolic genes are present in all studied species, with key catabolic and anabolic pathways often upregulated in the presence of another organism. These changes in gene expression fail to occur in conditions that will not lead to successful co-culture, suggesting they are essential for adapting to and surviving in the presence of others.

**IMPORTANCE:**

In 1882, Robert Koch and Fanny Hesse developed the agar plate, which enabled microbiologists to separate individual microbial cells from each other and create monocultures of a single strain of bacteria. This powerful tool has been used in the almost 150 years since to develop a robust understanding of how bacterial cells are structured, how they manage and process their information, and how they respond to the environment to produce behaviors that match their circumstances. We were curious about how the behavior of bacteria, as measured by their gene expression, changes between well-studied monoculture conditions and co-culture. We found that only specific growth conditions permit co-culture and that bacteria change their metabolic strategies in the presence of a partner.

## INTRODUCTION

Microbiology has developed a rich understanding of bacterial physiology, gene expression, and behavior through studies of bacteria grown in monoculture. But in the real world, bacteria grow in complex communities. Metagenomic studies have expanded our view of the identity and genomic content of bacterial species in many communities (1–4). Though metatranscriptomics (5, 6) and metaproteomics (7, 8) have questioned how these natural communities function, their scale and complexity preclude detailed investigation into the influence of community living on the behavior of individual species.

We set out to address this knowledge gap by asking what growth conditions enable simple two-species systems to form a stable co-culture with both members present for many generations. Then, we leveraged those co-cultures to ask how bacteria change gene expression when grown with another organism. By using only two species, we designed systems that, though far from representing the behaviors of bacteria in the wild, are tractable and suited for answering our question: What requirements do bacteria have, and what types of changes do bacteria make when placed in a community setting?

One fundamental question about co-cultures is as follows: how does the presence of one bacterium affect the growth of others? Microbes can have positive, neutral, and negative effects on the growth and survival of each other. For example, co-cultured lactic acid bacteria experience positive interactions in co-culture (9), and Coyte et al. observed rare, strong positive interactions in the human preterm infant microbiome (10). However, many interactions in systemic pairwise studies of gut microbiota include a negative growth effect on at least one member (11, 12).

The goal of our work was to understand what bacteria need to successfully create a community and how the presence in that community changes their behavior, as measured through gene expression. There are a few studies that investigate bacterial transcriptional and metabolomic responses during co-culture. In these systems, metabolic changes are common. For example, in a synthetic human gut tri-culture, metabolic profiling and RNA-sequencing showed competition for fructose and cross-feeding of formate (13). Similarly, *Roseburia intestinalis* co-cultured with *Bacteroides thetaiotaomicron* shifted toward acetate and lactate consumption while being inhibited by mucin sugars, while *B. thetaiotaomicron* responded by shifting to mucin sugar (14). In another study, *B. thetaiotaomicron* grown with *Escherichia rectale* in germ-free mice upregulated glycoside hydrolases to utilize mucosal glycans, while *E. rectale* decreased the expression of glycan-degrading enzymes and increased the expression of enzymes that generated butyrate from acetate (15).

We were interested to see if metabolic adaptation is a universal experience, so we designed two co-culture systems based on different pairs of organisms, *Pseudomonas aeruginosa* with *Escherichia coli* and *B. thetaiotaomicron* with *Lacticaseibacillus rhamnosus*. Though our goal was to focus on changes within individual bacterial species, not to model natural communities, we reasoned that these organisms might encounter each other in wastewater (16–18) or the mammalian gut (13–15). We defined growth conditions that support these two co-cultures. Transcriptomic analysis of bacteria in co-culture compared with monoculture revealed many changes in gene expression, including among anabolism- and catabolism-related genes. Surprisingly, these changes were not the same in all species, with key cellular processes such as translation or cell division being up or downregulated by different species in co-culture. We also observed that bacteria in conditions where they are less competitive can have muted gene expression responses, suggesting that the observed changes in transcriptional behavior are required for successful long-term coexistence with other bacterial species.

## RESULTS

**Fig 1:**
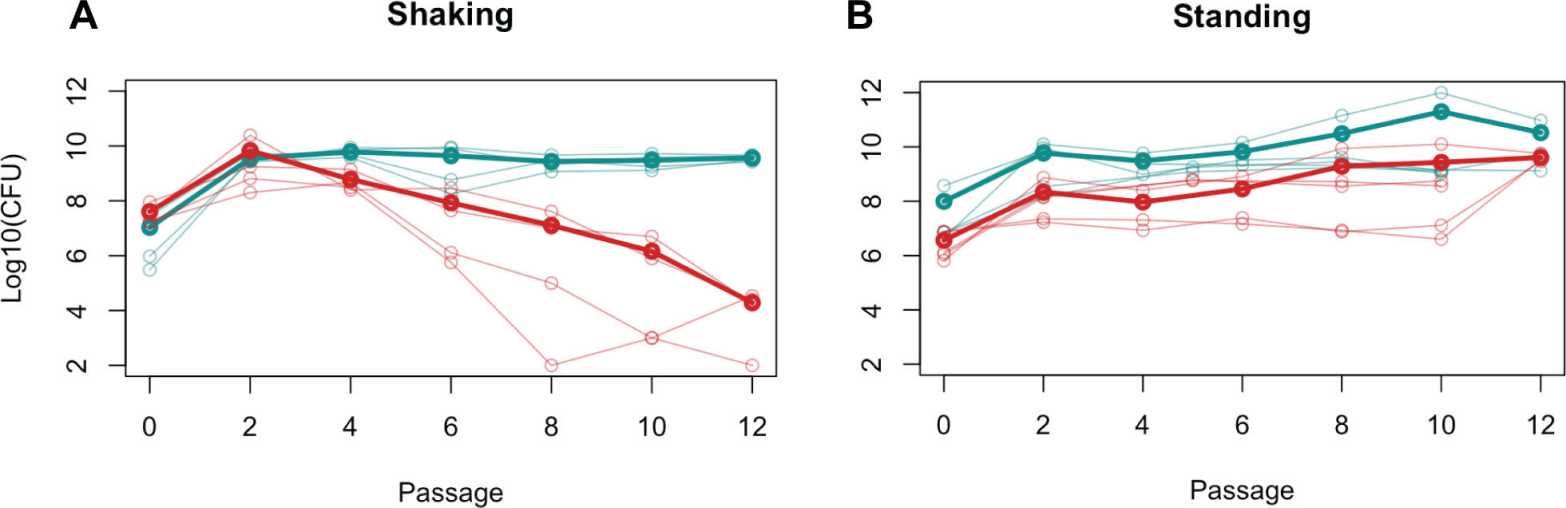
*E. coli* and *P. aeruginosa* co-culture succeeds in standing conditions. *E. coli* (red) and *P. aeruginosa* (blue) were co-inoculated into LB medium, incubated with shaking (**A**) or standing (**B**), and then back diluted into fresh medium every 24 hours. Colony-forming units (CFU/mL) were calculated at inoculation for passage 0 and after 24 hours growth for other passages. Thin lines show individual experiments, and thick lines show the average.

To investigate what changes bacteria undergo in a community, we created a two-species community of *P. aeruginosa* and *E. coli*. We defined a successful co-culture as a pair of species and a growth condition that meet two criteria. First, both species were present in the population at a reproducible ratio after many generations, and second, this ratio was the same across a wide range of inoculation ratios. *P. aeruginosa* and *E. coli* are naturally found in wastewater communities (19, 20) and were selected because they are well-studied, genetically tractable organisms. In the shaking culture, both species were initially present, as assessed by dilution plating on selection. But after many generations, achieved through serial passaging, *E. coli* was lost (Fig. 1A). These species have been previously shown to form biofilms together (21, 22), so we wondered if promoting biofilm production through static growth would produce successful co-culture. As shown in Fig. 1B, these conditions created a stable community that persisted for many generations. This co-culture was also robust to a wide range of inoculation ratios (Fig. S1).

### Gene expression changes in cell surface and metabolic genes accompany co-culture

**Fig 2:**
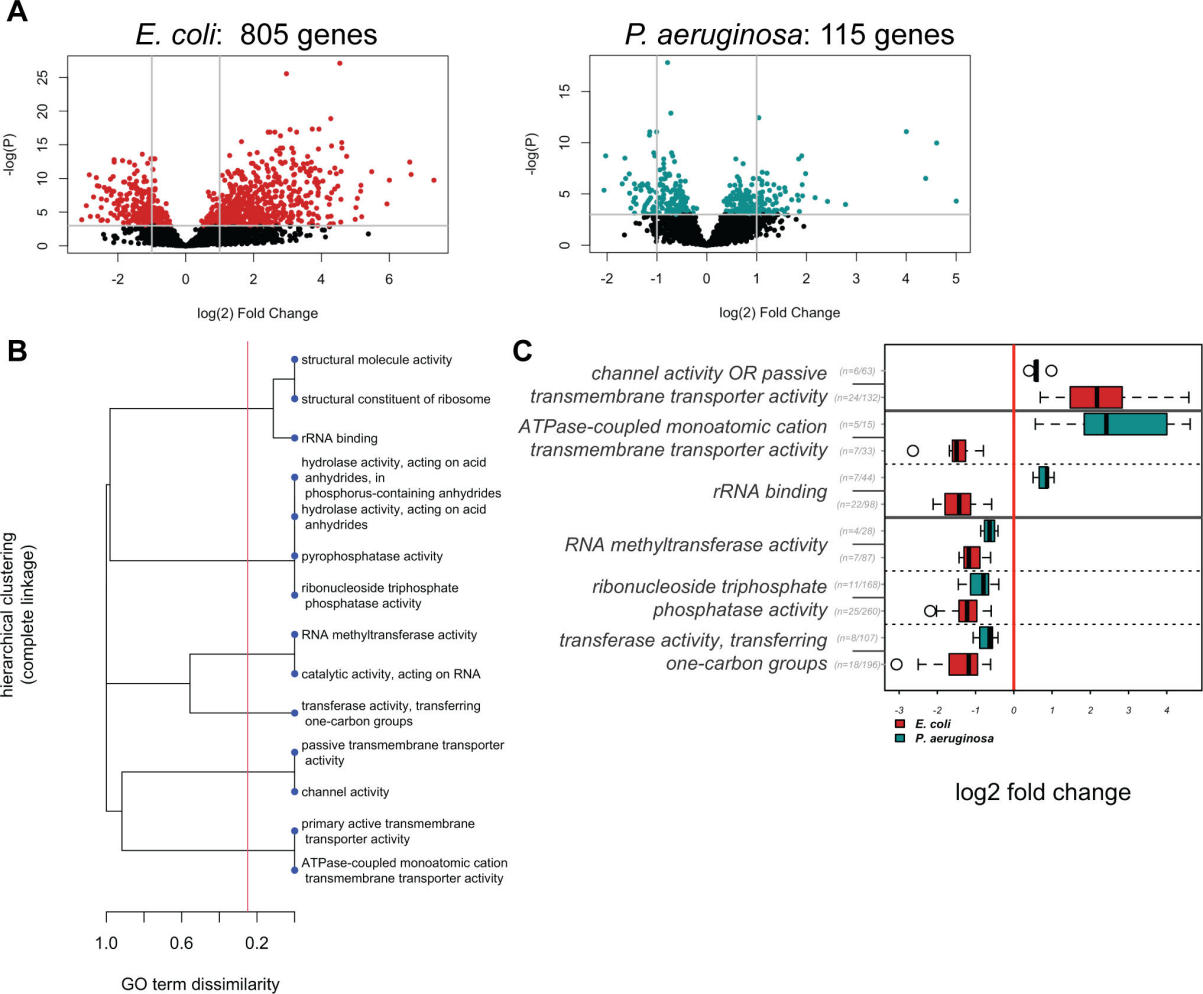
*E. coli* and *P. aeruginosa* have opposite transcriptional responses for metabolic genes in response to co-culture in standing conditions. (A) Volcano plots of *E. coli* (left) and *P. aeruginosa* (right) show the gene expression changes in response to co-culture, with genes expressed more abundantly in co-culture on the right. For reference, gray lines drawn at *P* = 0.05 and log_2_ fold change (l2fc) of 1 and −1. *E. coli* had 805 genes, and *P. aeruginosa* had 115 genes with *P* < 0.05 and |l2fc| > 1. (B) Hierarchical clustering of Gene Ontology (GO) terms based on the overlap of gene membership. The X-axis represents GO term dissimilarity, with clustering performed using complete linkage. A value of 1 denotes no shared genes, while a value of 0 denotes total overlap in genes between two GO terms. (C) The modeled log_2_ fold changes of genes in shared differentially regulated molecular function GO term clusters for *E. coli* and *P. aeruginosa* in response to co-culture. For each GO cluster and species, the ratio n, the number of differentially regulated genes in that cluster divided by the total number of genes in that cluster, is shown in gray. Outlier data points for each boxplot are indicated by open circles.

We sequenced RNA from cells grown in co- or monoculture under static, passaged conditions. As shown in Fig. 2A, we identified genes from both species that were differentially expressed in co-culture. Next, we determined which gene ontology (GO) terms were overrepresented among up and downregulated genes compared with their frequency in the genome (Table S1). We found the interpretation of the enriched GO terms complicated by the fact that many terms were redundant and that similar, overlapping groups of differentially expressed genes were driving the overrepresentation of multiple GO terms. Additionally, we wanted to compare the responses between species, so we combined GO terms with redundant gene membership within and across the two species into GO clusters (red line, Fig. 2B) and then compared the log_2_ fold change for the member genes of GO clusters that were enriched among differentially regulated genes in both species (Fig. 2C for the molecular function ontology, other ontologies in Fig. S2, full list of enriched GO terms Table S1).

GO cluster analysis suggests that in co-culture, both species remodeled their outer membranes by upregulating outer membrane porins and, in the case of *E. coli*, adding more fimbriae usher proteins (GO clusters channel activity and passive transmembrane transporter activity). In contrast, the species had opposite metabolic responses to co-culture. *E. coli* decreased the expression of the F_1_F_0_ ATP synthase, while *P. aeruginosa* increased the expression of this and other cation transporters (GO cluster ATPase-coupled monoatomic cation transporter activity). Additionally, *E. coli* decreased and *P. aeruginosa* increased the expression of the translation machinery (GO cluster rRNA binding). These opposite responses affected both catabolism (ATP generation) and anabolism (protein production).

### Oxygen availability determines *P. aeruginosa* and *E. coli* co-culture outcomes

**Fig 3:**
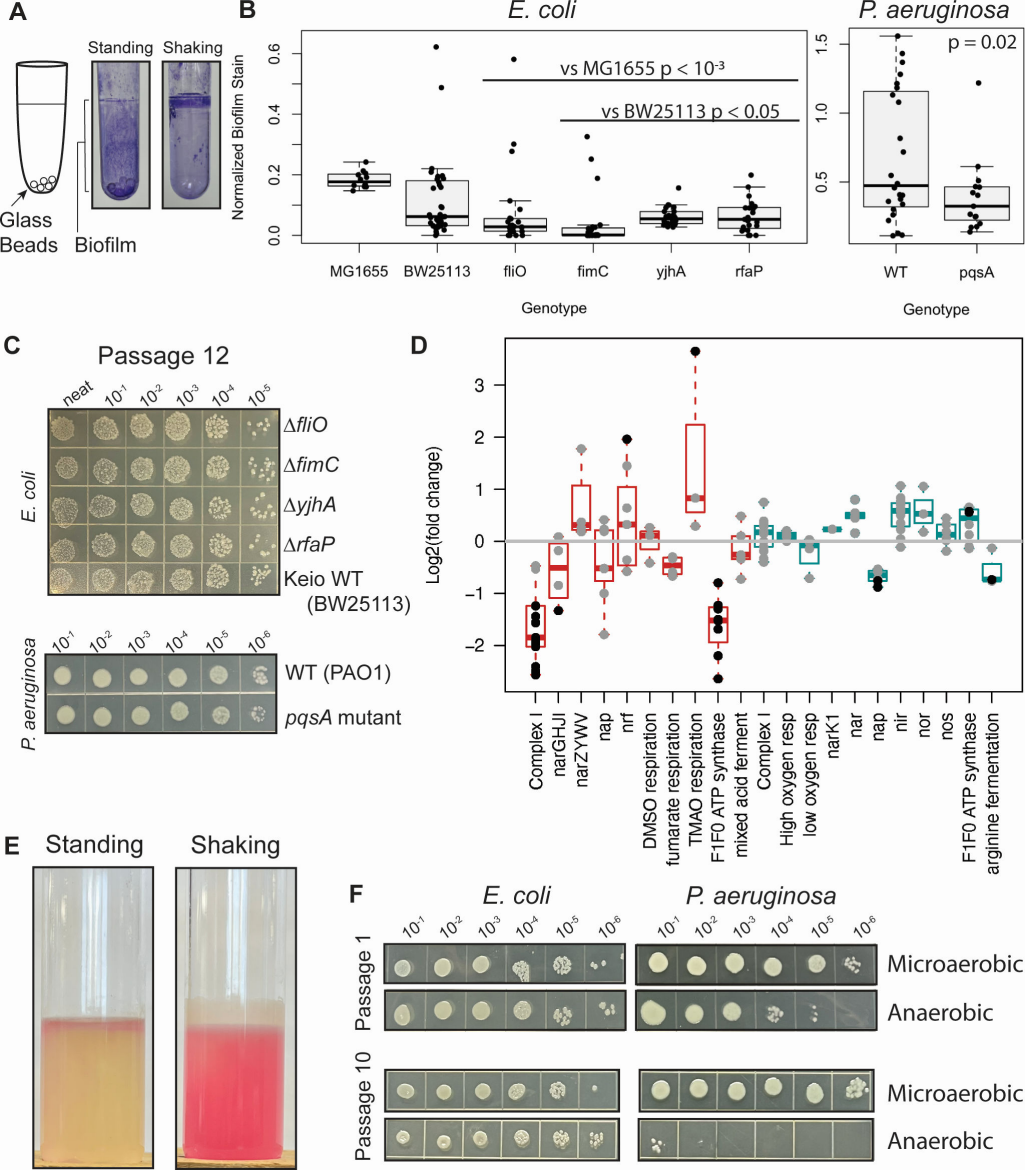
The presence of oxygen determines the success of *E. coli* and *P. aeruginosa* co-culture. (**A**) Crystal violet staining shows a biofilm on the surface of tubes grown in standing, but not shaking, conditions. (**B**) Biofilm production was measured as crystal violet stain normalized to cell growth (*n* = 12–36). *P* values from *t*-tests comparing *E. coli* mutant strains to MG1655 (*E. coli* WT strain used for co-culture) or BW25113 (background strain for *E. coli* mutants) and *P. aeruginosa* mutant to PAO1 (WT *P. aeruginosa*) are shown. (**C**) Dilutions of WT or biofilm-deficient mutants of *E. coli* or *P. aeruginosa* plated on selection for the indicated species after 12 passages of static co-culture with the WT of the other species. Similar results were achieved in three replicate experiments. (**D**) The log_2_ fold change (l2fc) upon co-culture for genes in various respiration and fermentation pathways. Red, *E. coli*; blue, *P. aeruginosa*. Genes whose l2fc adjusted *P* value is less than 0.05 are indicated in black. Genes used in this figure are listed in Table S2. (**E**) *E. coli* and *P. aeruginosa* co-culture grown in the Luria Bertani (LB) medium with resazurin in standing or shaking conditions. Oxygen, which turns resazurin to resofurin (pink), is present throughout the shaken tube and in the top layer of the standing tube. (**F**) Dilutions of *E. coli* and *P. aeruginosa* after one passage (top) or 10 passages (bottom) standing in air or in anaerobic conditions. Similar results were achieved in three replicate experiments.

Because static growth promoted both co-culture success and biofilm growth (Fig. 3A), we wondered if co-culture in static conditions changed the expression of biofilm genes. Indeed, *E. coli* upregulated the expressions of many such genes, as seen in its enriched GO terms (Table S1), but *P. aeruginosa* did not, explaining why these biofilm GO clusters do not appear in Fig. 2C, which displays only shared or complementary responses. To test whether *E. coli*’s biofilm response was essential for participation in static co-culture, we used *E. coli* mutants Δ*fliO*, Δ*fimC*, Δ*yjhA*, and Δ*rfaP*, which have biofilm formation defects due to disruptions in different pathways (23). Even though we observed reduced biofilm formation for all mutants (Fig. 3B), none had defects in co-culture participation (Fig. 3C). Though *P. aeruginosa* biofilm gene expression did not change in co-culture, *P. aeruginosa* may still contribute to biofilm production, promoting *E. coli* participation in standing conditions, so we assayed a transposon mutant of *pqsA* (24), an essential gene for PQS biosynthesis and biofilm formation in *P. aeruginosa* (25, 26). *pqsA* mutants also showed a biofilm defect (Fig. 3B), but no co-culture participation defect (Fig. 3C), suggesting that biofilm formation, while coincidental with co-culture success in standing conditions and upregulated in *E. coli* during co-culture, is not required.

Another difference between standing and shaking conditions is oxygen availability, so we looked at the expression of catabolic pathways in both organisms (Fig. 3D; Table S2). *E. coli* downregulated the expressions of key respiration genes, such as those encoding complex I and, as seen in the GO cluster analysis, the F_1_F_0_ ATP synthase. In contrast, the transcriptional response for respiration genes in *P. aeruginosa* was modest, with few genes differentially expressed. Standing co-cultures achieved mostly anaerobic conditions, with a small aerobic zone at the top, while shaken cultures were aerobic (Fig. 3E). This suggests that in microaerobic (standing) conditions, *E. coli* adjusted its metabolism to the other organism by relying less on respiration, while *P. aeruginosa*’s catabolic strategy remained unchanged in microaerobic conditions whether *E. coli* was present or not. We hypothesized that this adaptability may give *E. coli* an advantage in microaerobic conditions, which is missing in aerobic conditions. To test this idea, we further stressed respiration by growing standing co-cultures in an anaerobic environment. Though both species were present after the first 24 hours, by the tenth passage, *E. coli* remained and *P. aeruginosa* had been nearly eliminated from the culture (Fig. 3F). This suggests that aerobic conditions bias co-cultures toward *P. aeruginosa*, anaerobic conditions bias co-cultures toward *E. coli*, and the microaerobic conditions produced by cultures standing in air balance the two species over many generations.

### Metabolic gene transcription also responds to co-culture in a second system

**Fig 4:**
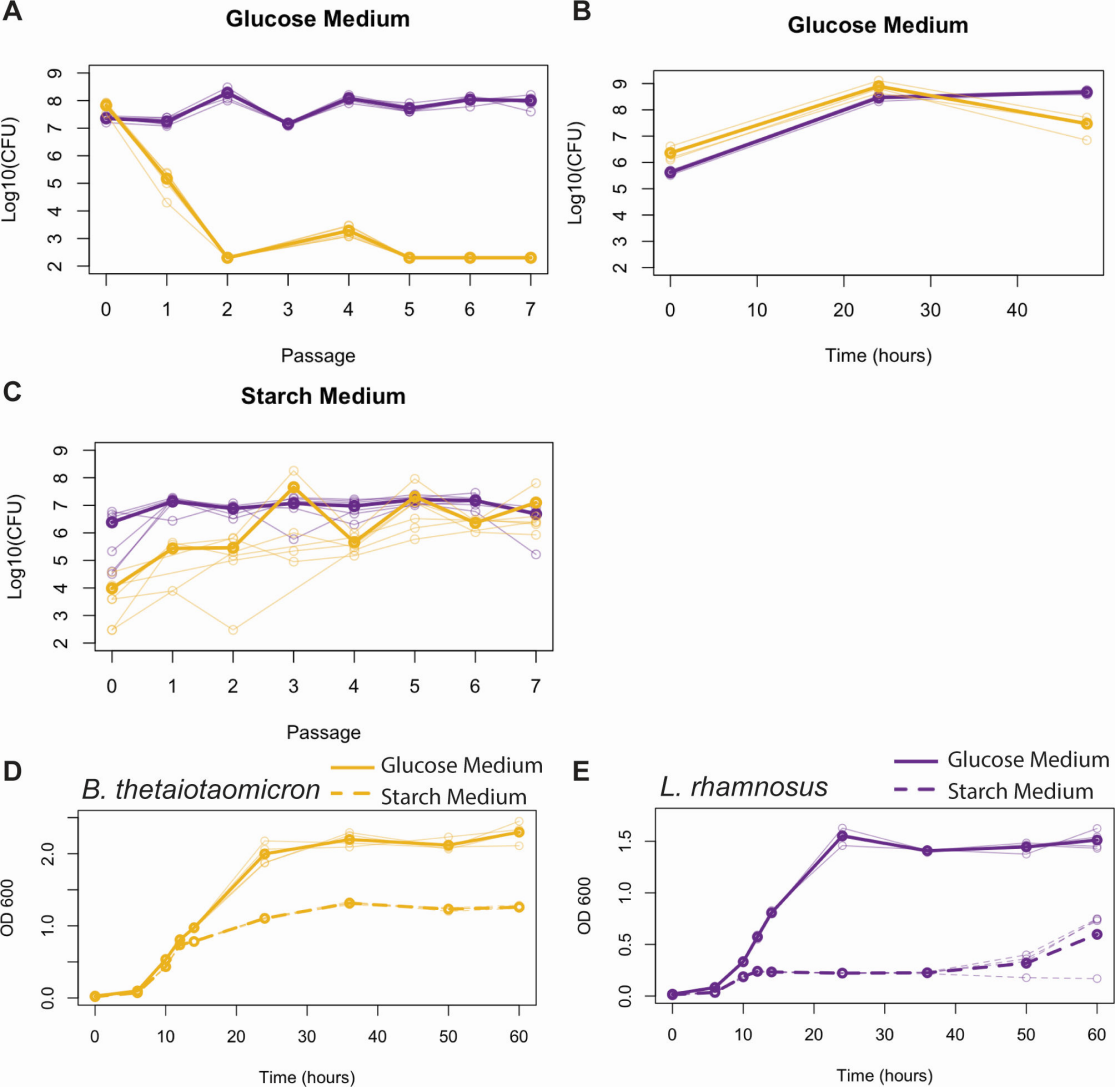
*L. rhamnosus* and *B. thetaiotaomicron* co-culture succeeds in the starch medium. *L. rhamnosus* (purple) and *B. thetaiotaomicron* (yellow) were co-inoculated into glucose (**A and B**) or starch (**C**) medium and then back-diluted into fresh medium every 48 hours for A and C. CFU/mL were calculated after 48 hours growth for A and C and at indicated time points for B. D) and E) The optical density at 600 nm of monocultures of *B. thetaiotaomicron* (**D**) or *L. rhamnosus* (**E**) in glucose (solid line) or starch (dashed line) media. Thin lines show individual experiments, and thick lines show the average. For A, B, and C, the limit of detection is 100 CFU/mL.

We were interested to see if similar gene expression changes accompanied co-culture in other pairings, so we grew *L. rhamnosus* and *B. thetaiotaomicron* in modified reinforced clostridial broth, which is a glucose-rich medium. Though this medium supported the growth of each species in monoculture, in co-culture, *B. thetaiotaomicron* was lost after several passages (Fig. 4A), with *B. thetaiotaomicron* CFUs even showing a decline within one culture throughout 48 hours (Fig. 4B). Because *B. thetaiotaomicron* can metabolize many sugars, including polysaccharides (27), we sought to bias growth toward this species by replacing glucose, which is usable by both species, with additional starch, which is only usable by *B. thetaiotaomicron*. Unlike the glucose medium, the starch medium supported both species in co-culture (Fig. 4C) and from many inoculation ratios (Fig. S3). Although the mean CFU/mL for *B. thetaiotaomicron* (thick line, Fig. 4C) appears to oscillate in these conditions, this is not true in all cultures (thin lines). Instead, rare outlier measurements increase the mean at some time points. This may be biological or an artifact of dilution plating.

In monoculture, both *B. thetaiotaomicron* and *L. rhamnosus* achieved stationary phase at a lower cell density in the starch medium than the glucose medium, but the effect was larger for *L. rhamnosus* (Fig. 4D and E). This could explain why *L. rhamnosus* outcompetes *B. thetaiotaomicron* in glucose but not starch medium. However, since *B. thetaiotaomicron* CFUs declined in glucose co-culture over 48 hours (Fig. 4B), *B. thetaiotaomicron* loss cannot be fully explained by its dilution across passages.

**Fig 5:**
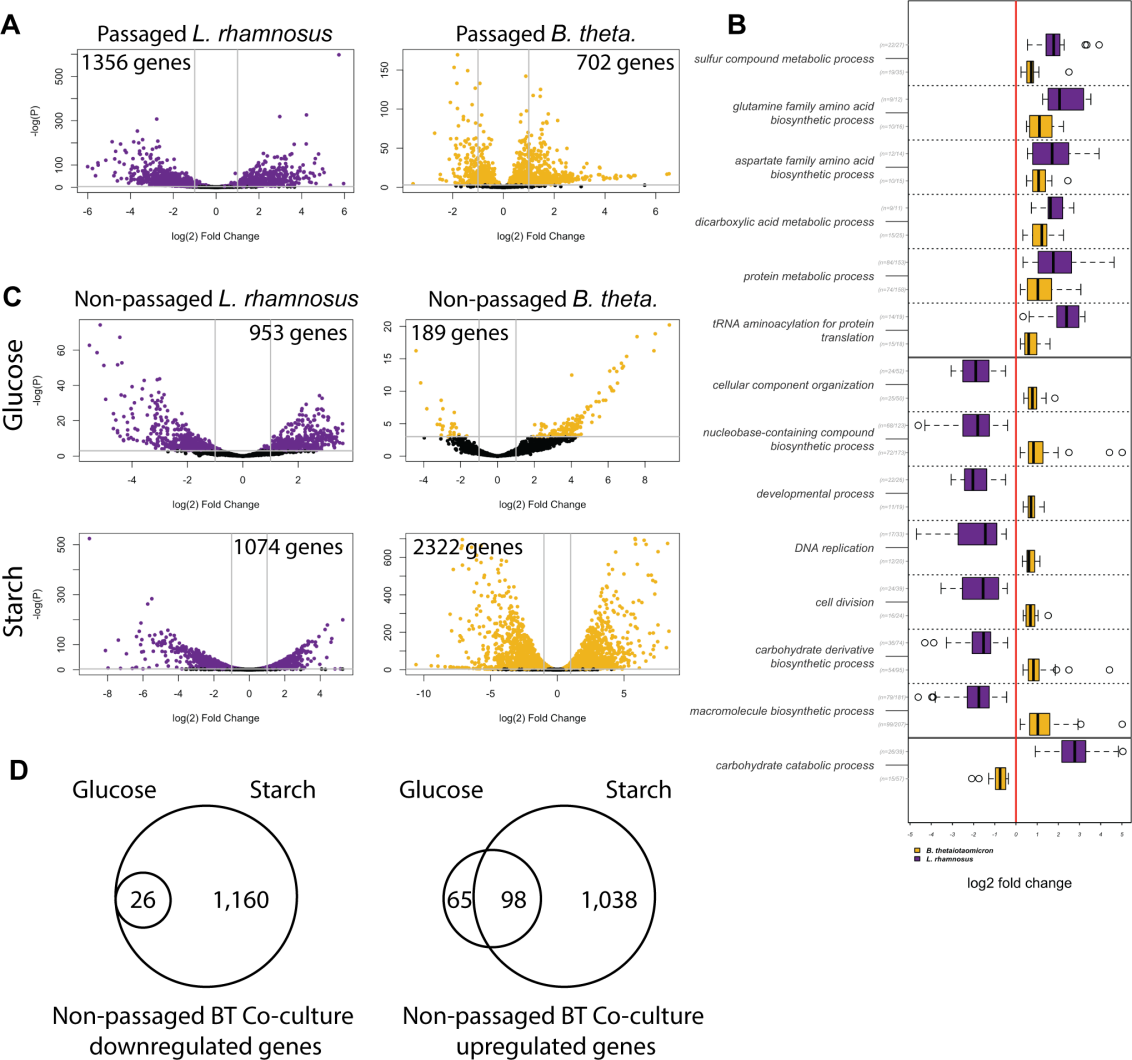
*B. thetaiotaomicron* exhibits large changes in gene expression in response to *L. rhamnosus* in starch, but not glucose medium. (A) Volcano plots of *L. rhamnosus* (left) and *B. thetaiotaomicron* (right) show the gene expression changes in response to passaged co-culture in the starch medium, with genes expressed more abundantly in co-culture on the right. For reference, gray lines drawn at *P* = 0.05 and log_2_ fold change of 1 and −1. *L. rhamnosus* had 1,356 genes, and *B. thetaiotaomicron* had 702 genes with *P* < 0.5 and |l2fc| > 1. (B) The modeled log_2_ fold changes of genes in shared differentially regulated biological process GO term clusters for *B. thetaiotaomicron* and *L. rhamnosus* in response to passaged co-culture. For each GO cluster and species, the ratio n, the number of differentially regulated genes in that cluster divided by the total number of genes in that cluster, is shown in gray. (C) Volcano plots show gene expression changes in response to co-culture in non-passaged conditions. *L. rhamnosus*, left; *B. thetaiotaomicron*, right; glucose medium, top; starch medium, bottom. The number of genes with *P* > 0.05 and |l2fc| > 1 is shown in each plot. (D) The muted response in glucose is a subset of the response in starch. Venn diagram showing the number of up and downregulated genes for each media condition shown in C.

We sequenced RNA from co- and monocultures passaged in the starch medium and applied our GO clustering to differentially expressed genes, as described above (Table S3; Fig. 5A and B for biological process ontology). For other ontologies, refer to Fig. S4). As in the *E. coli* and *P. aeruginosa* system, both species had enriched GO clusters among co-culture upregulated genes for protein-related metabolic processes, including amino acid synthesis and translation (GO clusters protein metabolic process and tRNA aminoacylation for protein translation). However, in the case of *B. thetaiotaomicron* and *L. rhamnosus*, both organisms upregulated these metabolic processes in co-culture. Both also upregulated redox enzyme production, including several acetate kinases, key enzymes in the generation of acetyl-CoA during sugar catabolism (GO cluster sulfur compound metabolic process).

Some processes were affected by co-culture in opposite directions in each species. Genes integral to glycolysis and the pentose phosphate pathway in the GO cluster carbohydrate catabolic process were enriched among upregulated genes for *L. rhamnosus*, but among downregulated genes for *B. thetaiotaomicron. B. thetaiotaomicron* upregulated genes that play a role in cell division, while *L. rhamnosus* downregulated these genes (GO clusters cellular compartment organization, developmental process, and cell division), including cell wall biosynthesis genes (GO cluster carbohydrate derivative biosynthetic process). Also upregulated in *B. thetaiotaomicron* and downregulated *in L. rhamnosus* were transcriptional genes, such as RNA polymerase and response regulators (GO clusters macromolecule biosynthetic process and nucleobase containing compound biosynthetic process).

### *B. thetaiotaomicron* lacks a robust gene expression response to *L. rhamnosus* in glucose medium

The RNA-seq results of both co-cultures suggest each organism adjusted its metabolism to accommodate the other. Since we observed that *E. coli* had an advantage in microaerobic concentrations where it was able to make bigger transcriptional adjustments to co-culture, we wondered if a muted transcriptional response to co-culture also characterized *B. thetaiotaomicron* in the glucose medium. We compared gene expression profiles from co- and monocultures of the two species in both media. Because *B. thetaiotaomicron* levels declined in the glucose medium co-culture (Fig. 4A and B), RNA for this experiment was collected from non-passaged cultures. As shown in Fig. 5C, *L*. *rhamnosus* experienced a large change in the gene expression due to the presence of *B. thetaiotaomicron* in both media conditions. Differentially expressed genes (Fig. S5; Table S4) included both catabolism- and anabolism-related genes and were similar in profile to the passaged response (Fig. 5B), except that cell division, which was downregulated in passaged co-cultures, was upregulated during the first culture. *B. thetaiotaomicron* also produced such a response in the starch medium. Surprisingly, co-culture in the non-passaged starch medium triggered more than three times as many genes to change expression than in passaged starch medium, suggesting that some genes respond only transiently soon after a new community member is added. In the glucose medium, however, the expression of few *B. thetaiotaomicron* genes was affected by *L. rhamnosus*, suggesting *B. thetaiotaomicron* co-cultured in glucose failed to make sufficient transcriptional changes. The muted response that *B. thetaiotaomicron* displayed in the glucose medium was mostly a subset of its successful response in the starch medium (Fig. 5D). Those genes upregulated in glucose but not starch included efflux pumps and nitrogen importers (Fig. S5B).

### Increases in cell size and investment in translation machinery accompany co-culture in *B. thetaiotaomicron*

**Fig 6:**
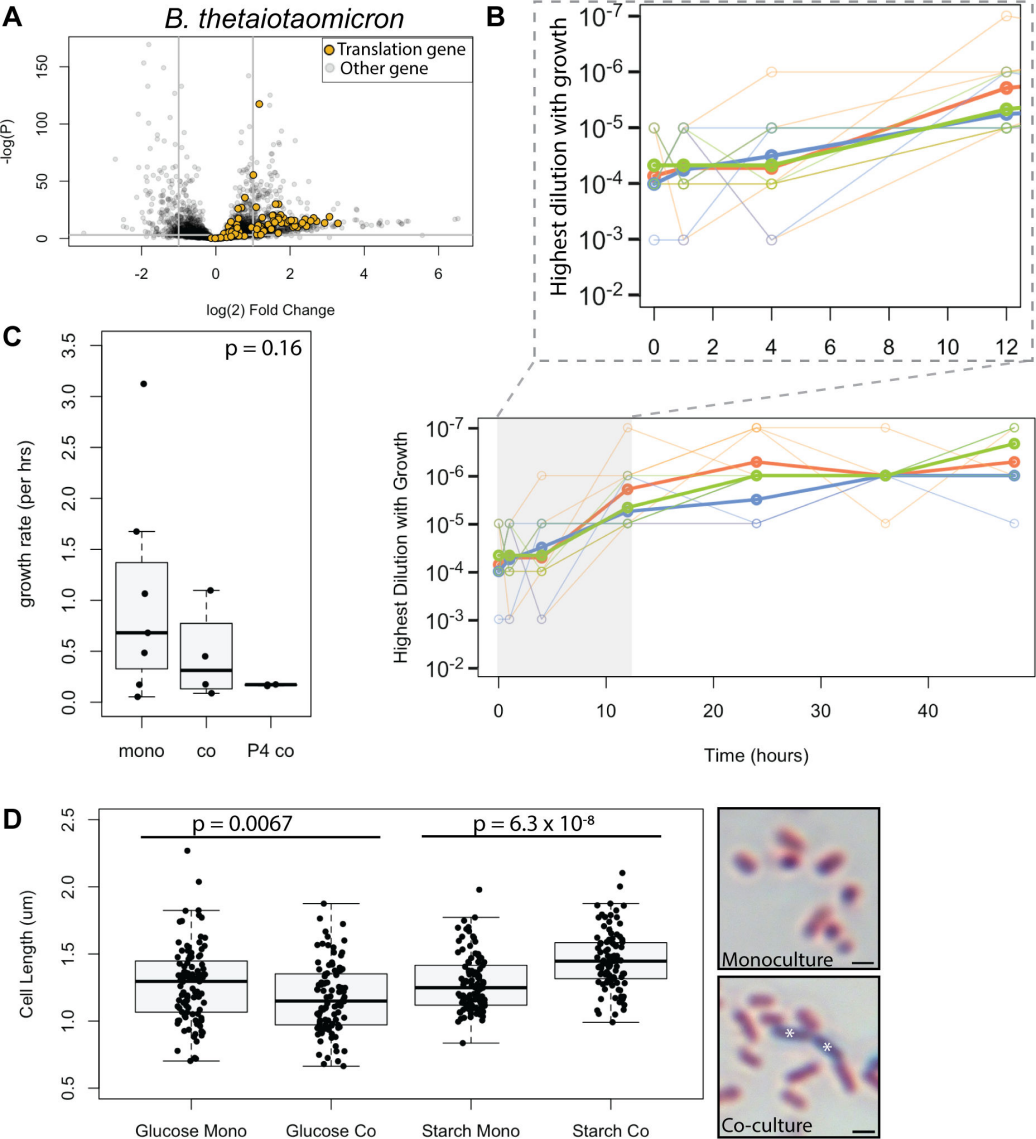
Co-cultured *B. thetaiotaomicron* cells invest more in translation machinery and are larger than monocultured cells. (A) The same volcano plot as in Fig. 5A, showing genes encoding ribosomal proteins and tRNA synthetases in yellow. Forty-eight out of 75 translation genes had *P* > 0.05 and |l2fc| > 1. (B) *B. thetaiotaomicron* cultures were grown in the starch medium under monoculture conditions (orange), 1st passage co-culture conditions (blue), or 4th passage co-culture conditions (green). At the indicated time points, the culture was serially diluted and plated. The highest dilution at which growth was still visible was recorded. Thin lines show individual experiments, and thick lines show the average. Inset expands the 0–12 hour portion of the graph. (C). The growth rate of cultures shown in B was modeled with the software growthcurver (ANOVA *P* = 0.16). (D) Co- and monocultures grown in glucose or starch media were gram-stained and examined under 100X DIC microscopy to determine *B. thetaiotaomicron* cell length (*t*-test *P* = 0.0067 for the glucose medium; *P* = 6.3 x 10^−8^ for the starch medium). Example microscopy images on the right. Scale bar = 1 µm, asterisks indicate gram-stained *L. rhamnosus* cells, which are excluded from the measurement of *B. thetaiotaomicron* lengths.

We were surprised by the expression of information processing genes, such as those encoding translational machinery, structural components of the ribosome, or tRNA ligases. This was surprising both because they were differentially regulated in co-culture in all organisms observed and because these genes are often thought of as housekeeping genes, whose expression does not change in response to the environment. In *E. coli*, ribosome components and translation machinery were downregulated during co-culture, but in other organisms, these GO clusters were enriched among upregulated genes. To better understand this observation, we looked at *B. thetaiotaomicron*, in which this pattern was pronounced (Fig. 6A; Table S3).

One situation in which cells would need high ribosome and tRNA ligase production is logarithmic growth. We wondered if *B. thetaiotaomicron* grows at different rates in co-culture and monoculture, which would result in RNA isolated from the same time point inadvertently comparing different growth phases. We grew *B. thetaiotaomicron* in the starch medium either by itself or with *L. rhamnosus* and enumerated *B. thetaiotaomicron* CFU (Fig. 6B). As shown in Fig. 6C, growth dynamics appeared the same between these conditions (ANOVA of modeled growth rates, *P* = 0.16).

We wondered if, instead of being in different growth phases, cells with different relative translation machinery gene expressions were reallocating resources for efficiency under resource-limited conditions. Cell size is one way that bacteria can control their growth efficiency, with larger cells being more efficient (28). Previous work has shown that *B. thetaiotaomicron* cells are longer under nutrient limitation conditions (29). We grew *B. thetaiotaomicron* with or without *L. rhamnosus* and then used a Gram stain to distinguish the two species under the microscope. As shown in Fig. 6D, co-cultured *B. thetaiotaomicron* were larger than monocultured cells in the starch medium (*t*-test, *P* = 1.4 x 10^−8^). This physiological adjustment was correlated not only with the presence of *L. rhamnosus* but also with successful co-culture since we observed the opposite effect in the glucose medium, which grew smaller co-cultured *B. thetaiotaomicron* cells (*t*-test, *P* = 0.0067). We measured no difference in the sizes of monocultured *B. thetaiotaomicron* cells between the two media (*t*-test, *P* = 0.8248).

## DISCUSSION

**Fig 7:**
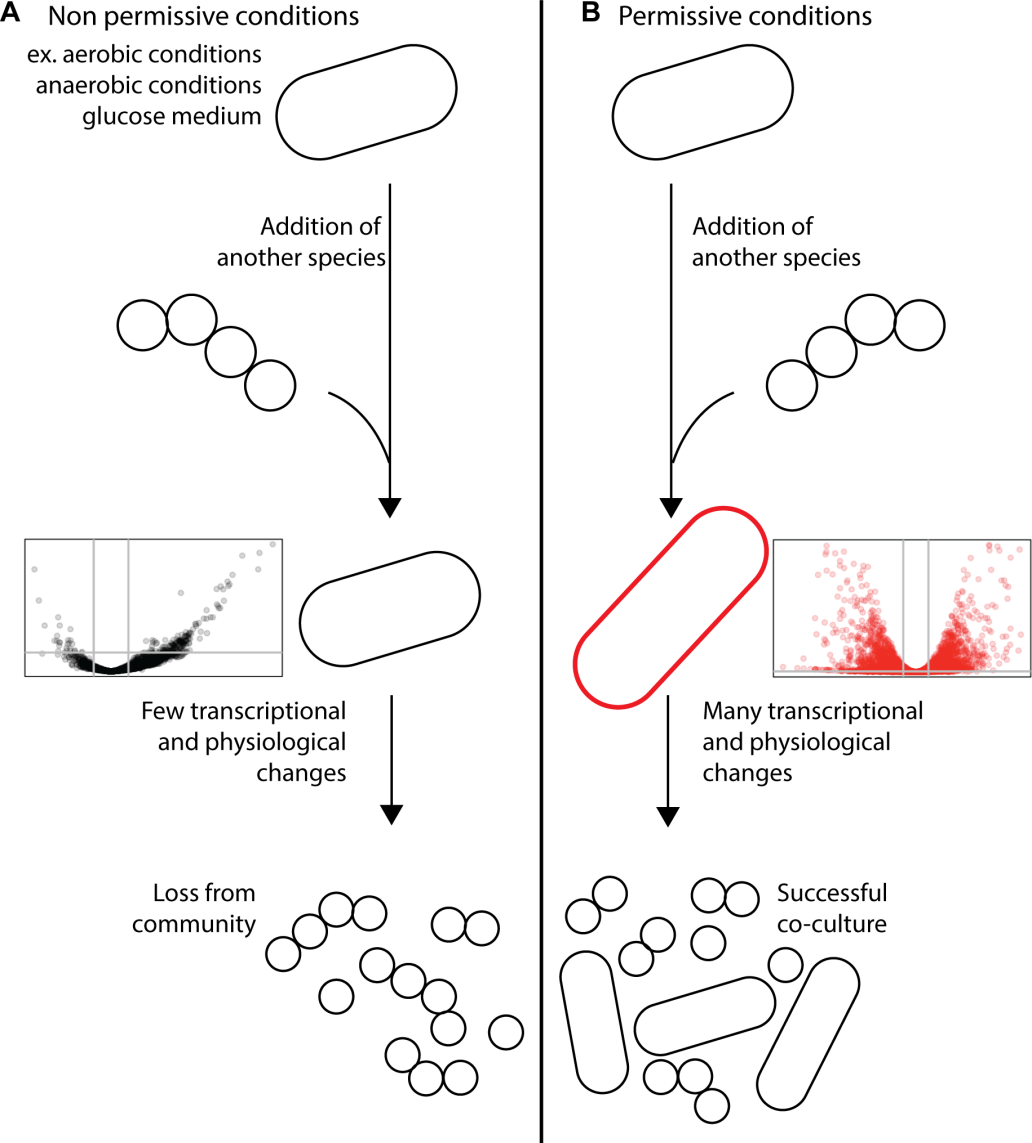
Under non-permissive conditions (**A**), some bacteria have little to no response to the presence of others and are unable to successfully participate in co-culture. Under permissive conditions (**B**), bacteria respond to the presence of other organisms by making transcriptional and physiological changes that promote successful co-culture.

In our two co-culture systems, we created conditions where both pairs of organisms grew at a stable ratio for many generations from different inoculation ratios by altering the nutritional resources or available oxygen. Through RNA sequencing, we showed that, as has been seen in other systems (13–15, 30, 31), changes in metabolism-related gene expression are common in co-culture. We propose a model where the capacity for gene expression changes and physiological responses to a partner organism determine co-culture success (Fig. 7). The direct evidence for this model comes from the *B. thetaiotaomicron* and *L. rhamnosus* co-culture. In this system, we saw *B. thetaiotaomicron* make an order of magnitude fewer gene expression changes in response to *L. rhamnosus* in the glucose medium, where it eventually failed, than in starch medium, where it eventually succeeded (Fig. 5C and D). This suggests that the permissive condition potentiated transcriptomic flexibility in *B. thetaiotaomicron*. Subsequently, we saw co-cultured *B. thetaiotaomicron* cells undergo changes in length, but again only in the starch medium (Fig. 6D). Though observed previously in *B. thetaiotaomicron* under starved conditions (29), the cause of this physiological response is unknown. We posit that this model could explain success and failure of co-culture in other systems. Though we did not measure gene expression in our *E. coli* and *P. aeruginosa* co-culture in non-permissive conditions, such as high or no oxygen, one can think of *P. aeruginosa* as being less competitive than *E. coli* in microaerobic conditions, which permits the success of *E. coli* in standing conditions. This could explain why P. *aeruginosa* had the fewest genes differentially expressed upon co-culture in permissive conditions among all organisms investigated here (Fig. 2A and 5A) and why *P. aeruginosa* appears to make few physiological adjustments to the presence of *E. coli*, as judged by the expression of core catabolic pathway genes (Fig. 3D).

### Changes in the expression of translation machinery are common in co-cultures

All species observed had changes in the expression of the core translation machinery such as tRNA synthetases and ribosomal proteins. Presumably, these reflect changes in the number of ribosomes produced. However, since our workflow measured the expression of protein-coding genes, the data on the amounts of tRNA and rRNA transcribed are missing from our analysis. One explanation for higher ribosome production would be an increase in the growth rate. Indeed, Morin et al. (30) observed overexpression of aminoacyl tRNA synthetase genes in co-culture in their system and attributed this to the co-cultures being in the log phase and the monocultures in the stationary phase. In our system, however, we showed that the growth phase was not affected by co-culture (Fig. 6B and C).

We argue that another situation which could result in translation gene upregulation is cells adopting an efficient form of growth that reduces the expression of unnecessary genes while retaining essential functions. This efficient growth would enable a species to maintain the carrying capacity of the environment, even as the overall resources are reduced due to the presence of a competitor. We observed no difference in CFU/mL for *B. thetaiotaomicron* between co- and monoculture (Fig. 6B). Instead, we saw a relative increase in the expression of translation machinery, suggesting a reallocation of cellular resources toward essential processes and a reorganization of cell size toward larger, more efficient cells.

Bacteria have been known to alter their physiology to adopt different growth efficiencies depending on the available resources (32, 33). Co-culture has affected the microbial cell efficiency, cell size, and carrying capacity, as noted in previous studies, with larger cells being more efficient (28). Starvation has also been observed to increase *B. thetaiotaomicron* cell size in monoculture (29). Taken together with our observations, this suggests that in co-culture with *L. rhamnosus*, *B. thetaiotaomicron* experiences nutritional stress, triggering transcriptional resource reallocation, and an increase in cell size that results in efficient cells that maintain the carrying capacity of the medium in the face of competition.

### Rich media: too much of a good thing?

Previous studies have investigated gene expression in co-cultures of *B. thetaiotaomicron* with different partners (14, 15, 34) or in other closely related pairs of gut microbes (13). In these examples, in agreement with our findings, differentially expressed genes are often found in catabolic pathways, such as sugar or other nutrient transporters and fermentation pathways, suggesting that obtaining carbon and reducing equivalents is a major source of competition in gut-simulation systems. Interestingly, Liu and colleagues showed that *B. thetaiotaomicron* and its partner organism in that study, *R. intestinalis*, impacted each other’s growth negatively in the presence of glucose, but positively once glucose had been consumed (14). Similarly, when comparing multiple pairs of bacterial and fungal species, Velez et al*.* (35) saw positive growth interactions in minimal, but not rich, media.

These examples agree with our findings that glucose medium did not support co-culture growth, but starch medium did. One might assume a richer medium would better support the growth of multiple species, but perhaps the absence of a highly prioritized carbon source, such as glucose, frees species to select alternative, complementary, carbon sources. We observed behavior supporting this idea when we compared the gene expression changes in response to *L. rhamnosus* co-culture between non-passaged *B. thetaiotaomicron* in glucose and starch media (Fig. 5C and D). In the starch medium, as we also saw in passaged starch co-cultures (Fig. 5B), *B. thetaiotaomicron* had enriched GO clusters such as carbohydrate catabolic process and macromolecule biosynthetic process among genes with differential expression. However, in the muted response to co-culture that *B. thetaiotaomicron* had in the glucose medium, where it was eventually lost from the community, the only enriched GO clusters among the few differentially expressed genes pertained to remodeling of the cell surface (Fig. S5; Table S4).

Gene expression changes related to cell surface remodeling were also observed in starch co-cultured *B. thetaiotaomicron*. These observations suggest that either the environmental triggers for metabolic gene expression changes fail to be sensed or the signal transduction is blocked, for example, by catabolite repression, in the glucose medium; but that cell surface remodeling changes are initiated or regulated via a different mechanism that can proceed in the presence of glucose.

We also saw that the availability of an essential component of catabolism, the electron acceptor, determined the success or failure of co-culture in *E. coli* and *P. aeruginosa*. In low oxygen conditions, the two organisms co-existed, but in high or no oxygen conditions, *P. aeruginosa* or *E. coli*, respectively, outcompeted its partner. *P. aeruginosa* is well-adapted to microaerobic conditions, with multiple cytochrome C oxidases that effectively deliver electrons from catabolism to oxygen at different oxygen concentrations and with the soluble redox-active molecule pyocyanin (36). Though these strategies may have been deployed in our microaerobic conditions, we did not see a change in their expression when *E. coli* was added to standing cultures.

*E. coli*, on the other hand, has many potential electron acceptors, including nitrate, DMSO, TMAO, and fumarate (37), while *P. aeruginosa* is limited in anaerobic conditions to denitrification or fermentation (36). Though the expression of terminal oxidases and fermentation genes were mostly unchanged in *E. coli* upon co-culture, the reduction in the expression of complex I, the F_1_F_0_ ATP synthase, and the upregulation of acid response genes (Table S1) suggest that *E. coli* may diversify catabolism to include fermentation in order to compete with *P. aeruginosa* in a way not available in oxygen. In our experiments, aeration was a condition that, like glucose, supported fast growth but not effective co-cultures.

### Clustering GO terms for easy comparison

Our analysis benefited from our clustering GO terms within and across species, which let us directly compare gene expression changes in response to co-culture in two organisms. When conducting RNA-sequencing analysis, one must compare two conditions only; but there are many circumstances, such as observing changes in more than one organism or observing multiple drug treatment groups where more than two conditions need to be compared. To support future research and facilitate similar analyses, we developed the R package “**GO_cluster,”** available at github.com/nvpinkham/GO_cluster. This package provides tools for analyzing, visualizing, and clustering gene ontology (GO) term relationships within and across species. Users can group GO terms based on shared genes within a single species and compare GO term similarities between species. Additionally, the package includes visualization features that plot the distribution of log fold changes of genes within representative GO terms. One caveat is that many non-model organisms contain a high number of hypothetical proteins not associated with GO terms, in which case GO_cluster may be less useful.

### What about transient interactions?

This joins a growing body of literature, cited throughout this work, that aims to understand how bacterial behavior changes in co-culture. Nevertheless, only a handful of co-cultures have been examined *in vitro*. One criterion we used to define co-culture was that conditions had to support both partner species’ growth indefinitely, but this may not be a dominant feature of natural communities. For example, Medlock et al. (11) systematically compared 15 pairs of microbes from altered Schaedler flora and found only one commensal relationship. The remaining 14 pairings had a negative growth impact on at least one partner. In the open systems that comprise natural ecosystems, transient interactions may dominate. It will be interesting to see if the behaviors described here for stable co-cultures also apply to dynamic microbial relationships.

## MATERIALS AND METHODS

### Strains, media, and growth conditions

The following strains were used: *E. coli* MG1655, *P. aeruginosa* PAO-1, *L. rhamnosus* (ATCC 7469), *B. thetaiotaomicron* (ATCC 29741); Keio collection (38) strains: JW5316 (Δ*fliO*), JW4279 (Δ*fimC*), JW5778 (Δ*yjhA*), JW3605 (Δ*rfaP*), and *P. aeruginosa* transposon mutant PW2798 (*pqsA*) (24). *E. coli* and *P. aeruginosa* were cultured on Luria Bertani (LB) medium (BD Difco DF0446-07-5) at 37°C and passaged by inoculating a fresh tube at 0.01 OD_600_ every 24 hours. When indicated, 1 ug/L resazurin was added. For shaking conditions, 250 RPM was used. For static conditions, 14 mL polystyrene tubes (Celltreat 230440) with five 4 mm glass beads and 5 mL media were used. Before sampling or passaging, cultures were vortexed to agitate beads and homogenize biofilm and planktonic fractions. *L. rhamnosus* was cultured on MRS (Research Products International L11000), MRCB (“glucose medium”), and MMRCB (“starch medium”) and *B. thetaiotaomicron* on MRCB and MMRCB (Table S5). Cultures were passaged every 48 hours by transferring 1/100^th^ culture to fresh media. *L. rhamnosus* and *B. thetaiotaomicron* were grown in an anaerobic chamber (Coy) at 37°C, as were anaerobic co-cultures of *P. aeruginosa* and *E. coli*. For solid media, 1.5% agar (Fisher BP9744500) was added. To enumerate CFUs, the following selective plating was used: to select only *E. coli*, LB with 20 ug/mL cefsulodin; to select only *P. aeruginosa*, LB with 50 ug/mL ampicillin; and to select only *L. rhamnosus*, MRS was incubated aerobically. To select only *B. thetaiotaomicron*, MRCB with 20 ug/mL ampicillin was incubated anaerobically.

### Crystal violet staining

For photograph, media and cells were decanted from grown co-culture. Tubes were rinsed with distilled water, and then crystal violet (0.1%) was added for one minute, after which the stain was decanted and rinsed away with distilled water. For quantitative assay, cultures were grown in 96 well plates, and then OD_600_ was measured with a plate reader (Tecan Sunrise). Media and cells were decanted, and plates were stained and rinsed as described above. Then, 30% acetic acid was added, and OD_595_ was measured with the plate reader. Normalized biofilm stain was calculated as OD_595_/OD_600_.

### RNA isolation and sequencing

Total RNA was isolated from cells pelleted from 1.5 to 3 mL culture with the Zymo Research Direct-zol RNA miniprep kit (R2053). Cells for passaged samples (*n* = 4 monocultures, *n* = 6 co-cultures) were harvested at passage end points for middle and late passages (8 and 12 for *E. coli* and *P. aeruginosa*; 4 and 6 for *B. thetaiotaomicron* and *L. rhamnosus,* respectively). For non-passaged samples (*n* = 4 monocultures, *n* = 4 co-cultures), cells were harvested at 0.5, 1, 12, and 48 hours of growth. RNA was subject to bacterial rRNA depletion (Azenta) and then sequenced at a read depth of 9–15 million 2 × 150 bp paired-end reads per sample on an Illumina sequencer.

### Data analysis

RNA sequences were pseudoaligned to the general feature format (GFF) files of *E. coli* (ASM584v2), *P. aeruginosa* (ASM676v1), *L. rhamnosus* (ASM615190v1), and *B. thetaiotaomicron* (ASM1413175v1) with Salmon (39). DESeq2 (40) was used to model the log fold change for each gene between compared conditions. GO terms enriched among lists of statistically significant up and downregulated genes were identified with clusterProfiler (41, 42). We observed substantial overlap in genes among GO terms identified with clusterProfiler’s enrichGo function. To make our results less redundant, we employed a pairwise cluster analysis with complete linkage to identify GO terms with 75% or more shared genes and then used the GO term with the highest proportion of significantly up or downregulated genes to represent the GO cluster. When the same GO terms were observed in two species, we took the mean pairwise distance between them for hierarchical clustering. The representative GO cluster was determined by choosing the GO term with the largest proportion of genes up or downregulated within it. To compare growth rates, growthcurver (43) was used to model growth curve parameters. Statistical analyses were performed in R. For *t*-tests and ANOVAs, Welch’s variant was used.

### Cell size measurement

Cultures were applied to a glass slide, dried by evaporation, and passed three times through a flame. To Gram stain, slides were exposed to crystal violet for 1 minute, iodine for 1 minute, 95% ethanol for 30 seconds, and safranin for 1 minute, with a distilled water rinse after each step. Slides were blotted dry and imaged with a 100X DIC lens on an Olympus BH2 microscope. Images were taken with the View 4K high definition microscope camera (I.Miller) and analyzed with ImageJ (44).

## Data Availability

RNA reads are available at the Sequence Read Archive PRJNA1107249. GO_cluster package and DESeqDataSet objects available at https://github.com/nvpinkham/GO_cluster.

## SUPPLEMENTAL MATERIAL

Supplemental figures jb.00075-25-s0001.docx Fig. S1 to S4.

Supplemental figure and table jb.00075-25-s0002.docx Fig. S5 and Table S5.

Supplemental tables jb.00075-25-s0003.xlsx Tables S1 to S4.

ASM does not own the copyrights to Supplemental Material that may be linked to, or accessed through, an article. The authors have granted ASM a non-exclusive, world-wide license to publish the Supplemental Material files. Please contact the corresponding author directly for reuse.
