# Supplementary material for: Community living causes changes in metabolic behavior and is permitted by specific growth conditions in two bacterial co-culture systems
Source: J Bacteriol. 2025 May 14;207(6):e00075-25. doi: 10.1128/jb.00075-25 (PMC12186488; doi:10.1128/jb.00075-25)
Supplement: Supplemental figures — Fig. S1 to S4. [file jb.00075-25-s0001.docx]

**Supplementary material**


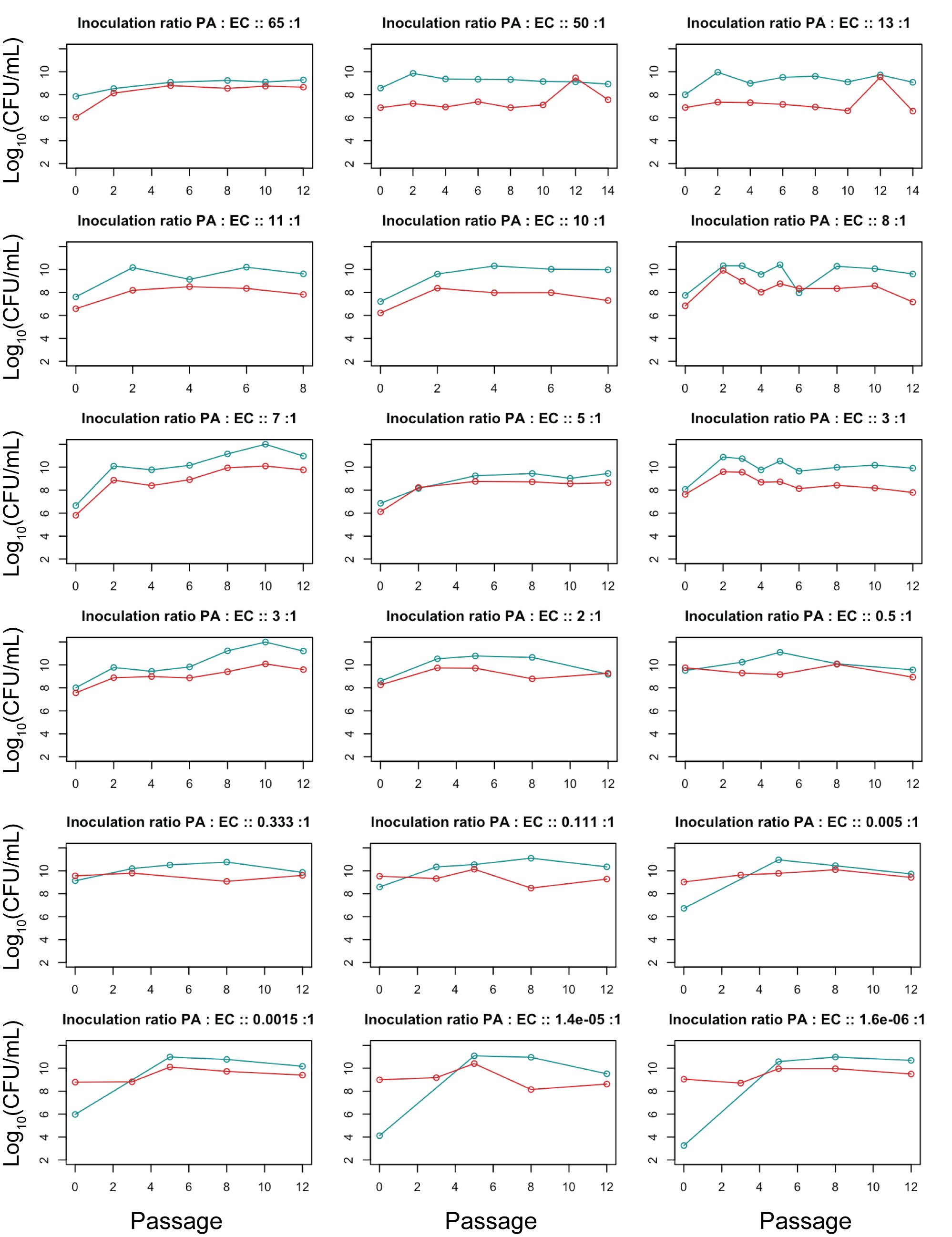


**Figure S1:** Multiple inoculation ratios of *P. aeruginosa* to *E. coli* result in successful co-culture after many passages. The Log_10_ of the CFU/mL of *P. aeruginosa* (blue) and *E. coli* (green) are graphed per passage. CFU/mL were assessed after 24 hours of growth except for passage 0, which was assessed at inoculation.


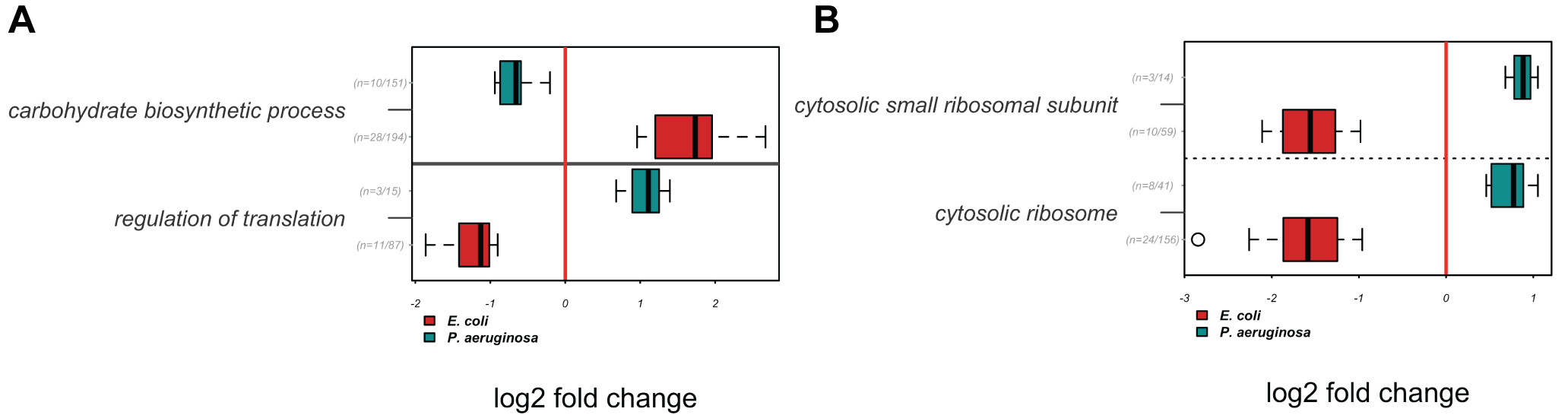


**Figure S2**: The modeled log_2_ fold changes of genes in shared differentially regulated GO term clusters for *E. coli* and *P. aeruginosa* in response to co-culture. A) Biological process GO term clusters. B) Cellular compartment GO term clusters. For each GO cluster and species, the ratio n, the number of differentially regulated genes in that cluster divided by the total number of genes in that cluster, is shown in gray.


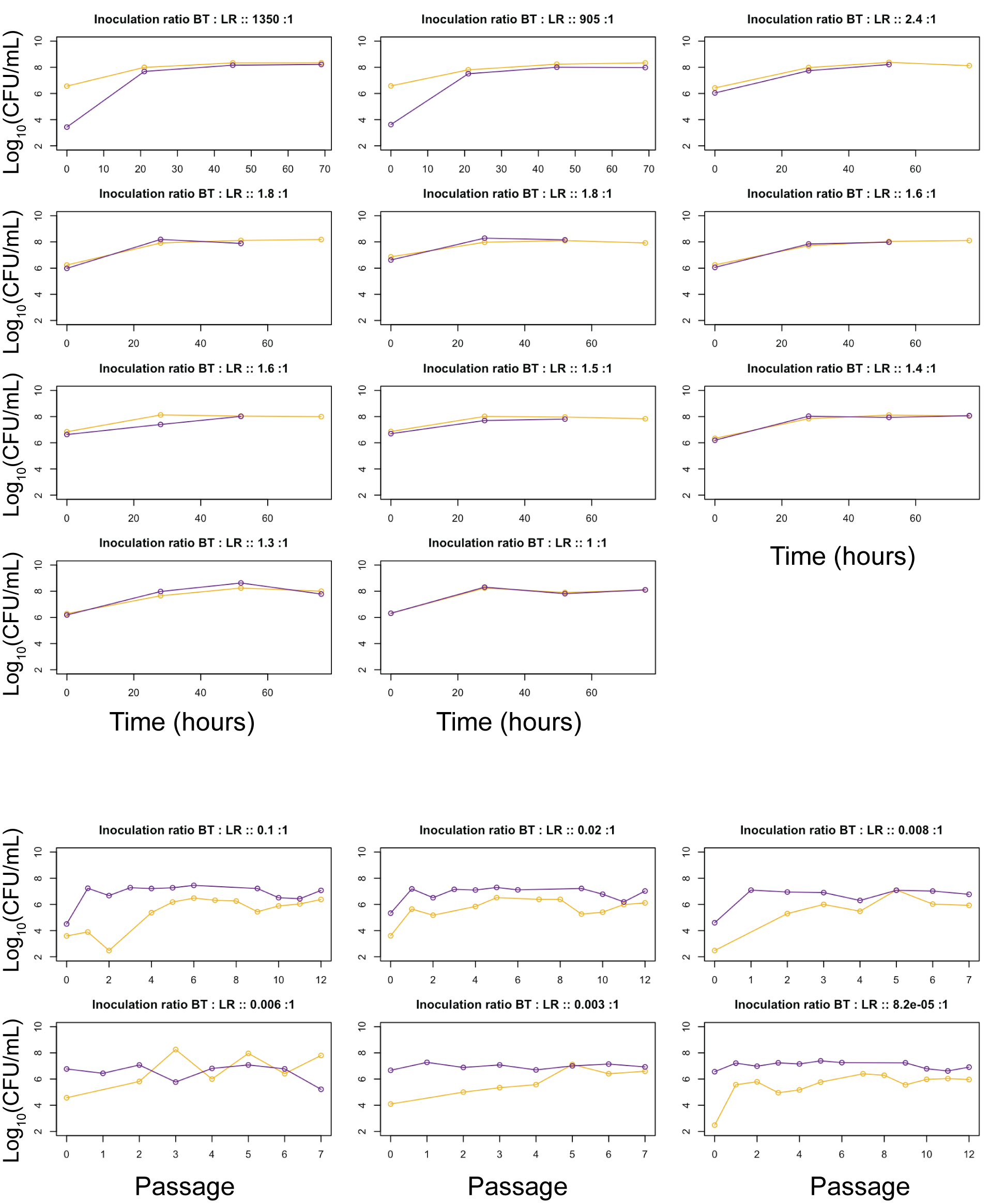


**Figure S3**: Multiple inoculation ratios of *B. thetaiotaomicron* to *L. rhamnosus* result in successful co-culture. The Log_10_ of the CFU/mL of *B. thetatiotaomicron* (yellow) and *L. rhamnosus* (purple) are graphed over time (top) or per passage (bottom). For passaged cultures, CFU/mL were assessed after 48 hours of growth, except for passage 0, which was assessed at inoculation.


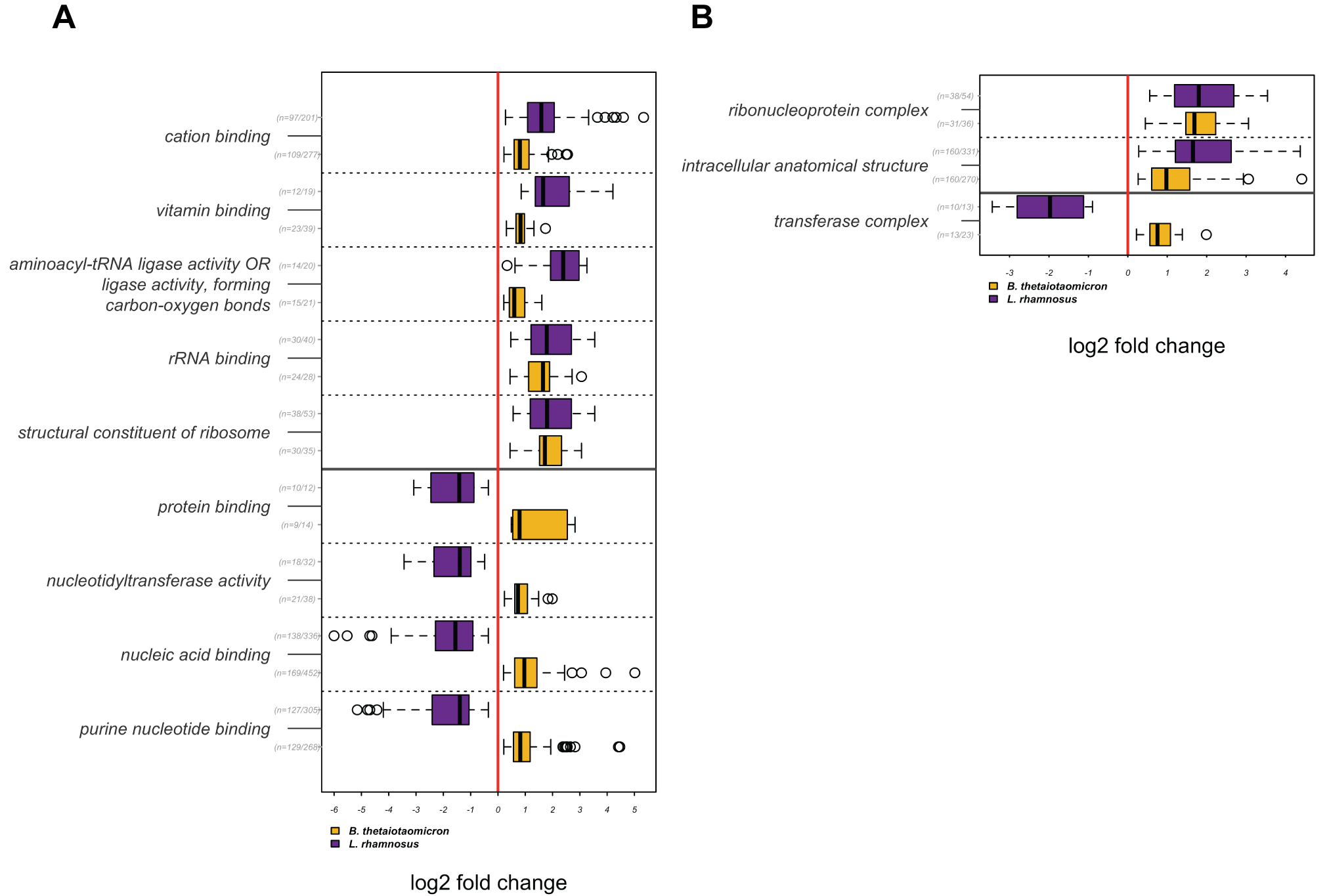


**Figure S4**: The modeled log_2_ fold changes of genes in shared differentially regulated GO term clusters for *B. thetaiotaomicron* and *L. rhamnosus* in response to co-culture. A) Molecular function GO term clusters. B) Cellular compartment GO term clusters. For each GO cluster and species, the ratio n, the number of differentially regulated genes in that cluster divided by the total number of genes in that cluster, is shown in gray.
