# Supplementary material for: Community living causes changes in metabolic behavior and is permitted by specific growth conditions in two bacterial co-culture systems
Source: J Bacteriol. 2025 May 14;207(6):e00075-25. doi: 10.1128/jb.00075-25 (PMC12186488; doi:10.1128/jb.00075-25)
Supplement: Supplemental figure and table — Fig. S5 and Table S5. [file jb.00075-25-s0002.docx]

**Supplementary material**


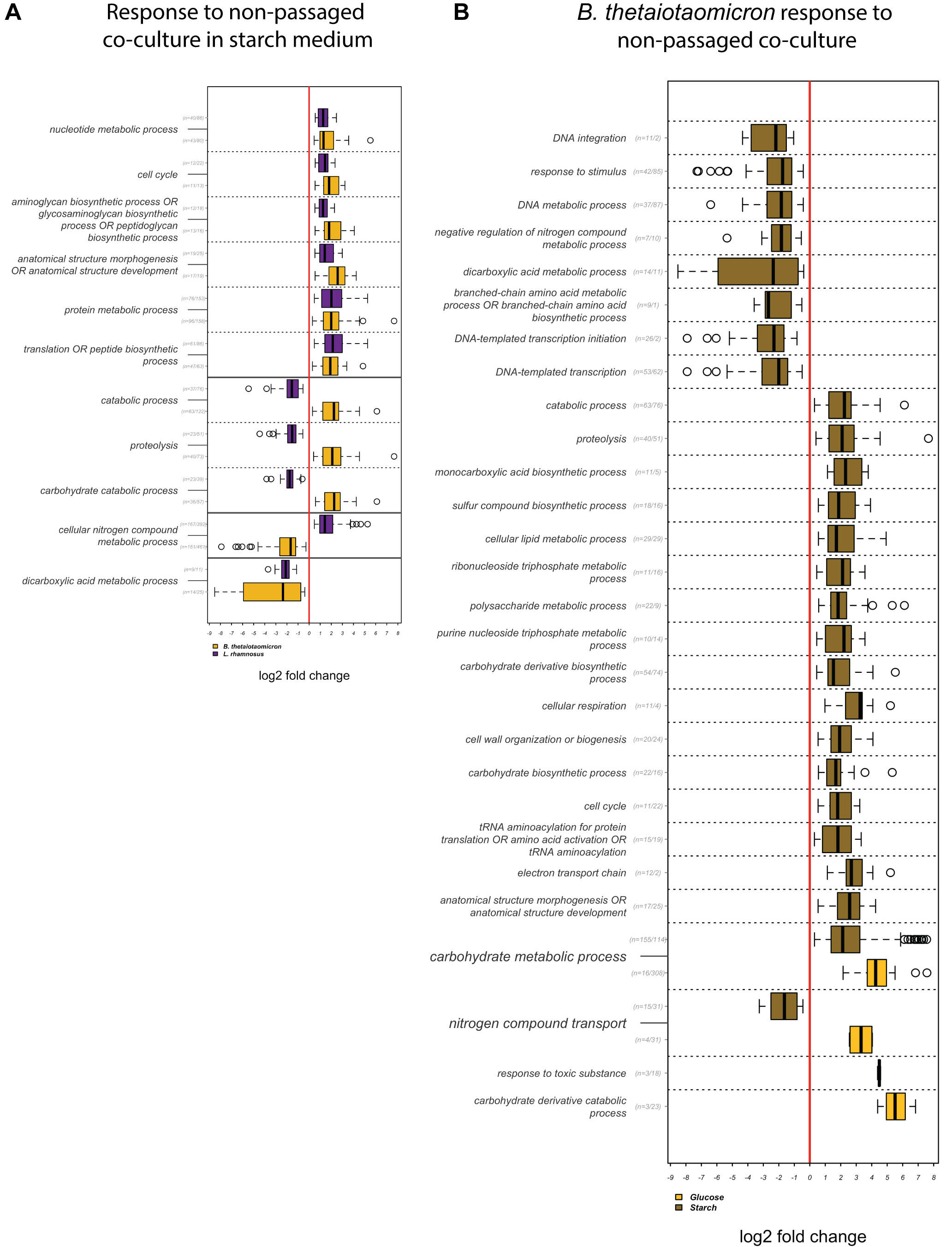


**Figure S5**: Starch media conditions are required for full transcriptional response to co-culture for *B. thetaiotaomicron*. A) The transcriptional response to co-culture in non-passaged starch medium is similar to that in passaged starch medium (Figure 5B), except for *L. rahmnosus* cell division genes. The modeled log_2_ fold changes of genes in shared differentially regulated biological process GO term clusters for *B. thetaiotaomicron* and *L. rhamnosus* in response to co-culture in non-passaged starch medium. B) *B. thetaiotaomicron* response to co-culture in glucose medium is a subset of its response in starch medium. The modeled log_2_ fold changes of genes in differentially regulated biological process GO term clusters for *B. thetaiotaomicron* in glucose (light yellow) or starch (dark yellow) media. For each GO cluster and species, the ratio n, the number of differentially regulated genes in that cluster divided by the total number of genes in that cluster, is shown in gray.

**Supplemental table 5**

| **Reagent** | **Amount per Liter in MRCB (Glucose medium)** | **Amount per Liter in MMRCB (Starch medium)** |
| --- | --- | --- |
| Peptone | 10 grams | 10 grams |
| Beef Extract | 10 grams | 10 grams |
| Yeast Extract | 3 grams | NA |
| Dextrose | 5 grams | NA |
| Starch | 1 gram | 2 grams |
| NaCl | 5 grams | 5 grams |
| L-cysteine | 0.5 grams | 0.5 grams |
| sodium acetate | 3 grams | 3 grams |
| NaOH | Enough to bring the pH to 6.8 | Enough to bring the pH to 6.8 |
| Agar (for solid medium) | 15 grams | 15 grams |
| Vitamin K1 (0.2 uL/mL) | 500 uL | 500 uL |
| Hemin (5 mg/mL) | 500 uL | 500 uL |
